# Round the Hospitals

**Published:** 1920-10-02

**Authors:** 


### ROUND THE HOSPITALS.

The new edition of the Register of Nurses maintained by the College of Nursing is a thoroughly live and progressive bit of work. It carries the record of registration to the end of March and includes mention of the training-school, certificates issued by certain public bodies, and honours conferred by the Sovereign. Extra distinctions and qualifications of this nature can be inserted if notice is given to the Registrar by the end of this year for the next edition, and for this privilege a fee of 5s. is payable. Notice of change of name or address must be given by January 31. A great part of the value of such productions as this depends on their being kept up to a right pitch of accuracy, and members ought to co-operate with the registrar by giving prompt notice on their own account and stimulating others to do the same. It has often happened that the only information procurable of the death of a nurse has come from one of her comrades. The Register is a clearly printed volume which runs to 523 pages; it averages some thirty-three names to a page, and we deduce the fact that exclusive of deaths and resignations the entries number over 17,000 nurses—a highly respectable total.

A severe strain has been put on the resources of the Metropolitan Asylums Board, so far as labour is concerned, by the autumn rise in fever cases. Their recruiting department is highly developed, and the Ministry of Labour, through the Labour Exchanges, passes many good candidates through to the service of the hospitals; but in some institutions a rush of cases has caused considerable pressure on a staff which has not been able to expand sufficiently in the time. The regulations which provide for overtime pay undoubtedly tend to alleviate these heavy spells, and the spirit of devotion is not discouraged in a nurse, because she is handed a comfortable addition to her salary as a result of surrendering some part of her leisure to the call of necessity.

In America they believe in direct appeal from the training-schools to women of age to begin training as nurses. Nightingale postcards are being circulated by the thousand to girls whose names have been obtained from the principals of high-schools. The cards contain such admonitions as, "Know the Joy of Service—Be a Nurse," interspersed with pictures of the hospitals. Thousands of leaflets and bulletins have also been put in circulation, lectures are being given at the high-schools by probationers in uniform, who give an account of their own doings, and the newspapers have been engaged to further the campaign by publishing inside information. All this is, at any rate, attracting general attention to the career of nurses, and instead of being exposed to the public as a sordid, ill-paid, and hopeless occupation, divided into the down-trodden and the down-treaders, nursing in its brighter and deeper aspects is kept well in view. That the campaign will react for good on the training-schools is already evident. For when all combine in such a publicity movement there is inevitable competition to offer a healthy and thoroughly attractive term of training to the students who flow in.

A remarkable gathering is taking place this week to give a hearty send-off to twenty-four missionary nursing sisters on the eve of their departure for distant lands. It is an occasion to warm the heart and inspire the imagination. To read over the list of destinations is to be aware that this is in truth empire-building in its highest sense. To places in India, Beluchistan, Egypt, Ceylon, Newfoundland, East and Central Africa, Nigeria, and other British possessions the nurses follow the flag, while their presence brings light and solace to remote regions in China, Madagascar, and on the Belgian Congo. To all the pioneers, destined, we do not doubt, to build more enduringly than they dream, we offer a message of sympathy and God-speed. The Secretary of the Nurses' Missionary League is Miss H. T. Richardson, 52 Lower Sloane Street, S.W. 1.

A luncheon party of rather a remarkable order is to be held at the Fishmongers' Hall on October 19, when a hundred women will rally to support the Royal Free Hospital School of Medicine for women, each bringing a male guest. The Committee includes, among many well-known names, that of Mrs. Massey Lyon (chairman), Lady Londonderry, Lady Ampthill, Lady Bland-Sutton, Miss Aldrich Blake, Mrs. Alice Perrin, Dr. Mary Scharlieb, Lady Cowdray.

It will be a calamity if no steps are taken during the Red Cross rally, October 9 to 17, to enlist women for service as whole-time nurses in the hospitals. A good proportion of the girls who take up Red Cross work could be drawn into higher service for the sick if the needs of the profession and its many openings to public service were brought to their notice. There will be gatherings all over the country to talk about future developments of Red Cross work, and it ought to be the rule to invite a trained sister in uniform to give a few minutes' talk at each meeting about the call for probationers.

The Lincoln Board of Guardians is being urged by the Ministry of Health to provide a new infirmary for the sick. The Guardians have attempted to evade this obligation by boarding out the imbeciles and constructing cubicles and a new block for the nurses, but still the Ministry is not satisfied. The Guardians must build, and find the money. They have referred the problem to the Housing Committee, and in due course, no doubt, the sick poor of Lincoln will have their infirmary.

**ROUND THE HOSPITALS—(*continued*).**

A VICTORY Ball is to be held, as before, on Armistice Day, November 11, at the Albert Hall, and this year the proceeds are to be divided between Queen Victoria's Jubilee Institute for Nurses and the British Red Cross Society.

---

OUR readers will remember the tragic story of the 800 Russian children discovered by the American Red Cross running wild in Siberia, half naked, living on roots and berries. They had been sent from Moscow and Petrograd by their parents to the Ural mountains for greater safety, and were there left deserted after the revolution. The American Red Cross founded orphanages and undertook their care. Lately they have brought some of them to Vladivostock and thence to Europe, while unremitting efforts are in progress to restore them to their relatives, many of whom are now refugees. Lists have been prepared giving the children's names and their last address at home, and these can be obtained by any likely to throw light on the matter from the League of Red Cross Societies, 2 Rue de la Soie, Geneva.

---

THE matron and nurses at Abergavenny Workhouse, who have taken the very proper course of resigning in consequence of the Guardians' refusal to raise their salary or grant them an interview, must be rather astonished at the publicity accorded to their action. In this transition time, the only dignified course for nurses dissatisfied with the conditions under which they are working is resignation, but we trust that in this instance sympathisers will see to it that other work is offered to the retiring nurses.

---

LADY BRADFORD'S modest little book entitled "A Hospital Letter-Writer in France" (Methuen & Co., 5s. net.), deserves a place in every hospital library, if only for its simplicity, good humour, and happy appreciation of the honour of serving. The author was already well versed in hospital visiting before, at the end of 1914, she followed her husband, Sir John Rose Bradford, to Boulogne, where his official duties as Consulting Physician to the British Expeditionary Force kept him engaged. From that time during the whole war, and some months after the signing of the Armistice, she devoted herself entirely to visiting the men, writing letters for them, and supplying them with stationery and comforts. It was work undertaken in the best possible spirit and carried through with evident disregard of self and with entire efficiency. The men accepted her "as their own private property." She was "Mother" and "Little Mother" to them all, and the only dissatisfaction caused was when some jealous sweetheart from afar mistrusted her attractions. Many of our readers will remember her presence in the wards and wish that other hospital visitors could learn the secret of her tireless, warm-hearted, and always welcome attentions to the patients.

---

A PAINFUL case has occurred at the Sleaford Workhouse, and has very properly been brought to the notice of the Sleaford Guardians by the House Committee. This Committee has passed a resolution recommending the Guardians to ask for the resignation of the master and matron (Mr. and Mrs. Parker), on the ground that a dying inmate of the workhouse was left from 8 P.M. to 7 A.M. without attention, and that she suffered from bedsores. At an inquiry held to investigate the matter it transpired that it was not the custom to engage a night nurse, although the medical officer had power to do so. The officials stated that "the patients helped each other," and were not convinced that this unhappy woman needed a night nurse "more than any other patient." They had "tried to manage as they did before." The Committee, however, took a different view of the necessities of the sick, and insisted on the engagement of a night nurse. The Board finally approved the action of the House Committee, but, in lieu of the first-named resolution, decided there had been "serious neglect by responsible officials, who should be requested to be more careful in future in similar cases." We trust this means that a night nurse will be engaged.

---

DURING the war there was a good deal of unfavourable comment on the employment of British nurses for the care of Indian soldiers. The system has now been introduced in India and has met with the approval of the native community, at any rate as a prominent Mussulman gentleman who has been visiting the military hospitals on the North-West Frontier has a long article in the leading newspaper, in which he expresses his favourable impression of the tidiness and general cheerfulness of the wards, which he attributes in great part to the presence of British nurses for nursing the sick Indian soldiers and followers. He comments on the admirable ablution annexes to the wards, with plentiful supplies of hot and cold water, and points out that each ward has electric light and fans! There is, moreover, a plentiful supply of ice, so that these hospitals are immeasurably superior to the ordinary station hospitals in which the British troops are treated "down country."

---

THE Stourbridge and Halesowen Hospital Committee is between the devil and the deep sea, for while the chairman, Mr. J. A. Tate, records that the rates at Halesowen are 17s. in the £, and are expected to rise to 21s., the matron of the Isolation Hospital cannot secure a staff at the present rate of salaries. The medical superintendent supports her contention that the salaries are insufficient, and the committee has at length decided to adopt the matron's proposal of an all-round increase of £10 a year. The new scale is to come into force at once.

